# Enhanced Therapeutic Efficacy of iRGD-Conjugated Crosslinked Multilayer Liposomes for Drug Delivery

**DOI:** 10.1155/2013/378380

**Published:** 2013-04-15

**Authors:** Yarong Liu, Man Ji, Michael K. Wong, Kye-Il Joo, Pin Wang

**Affiliations:** ^1^Mork Family Department of Chemical Engineering and Materials Science, University of Southern California, 3710 McClintock Avenue, RTH509, Los Angeles, CA 90089, USA; ^2^Department of Biochemistry and Molecular Biology, University of Southern California, Los Angeles, CA 90033, USA; ^3^Division of Medical Oncology, Norris Comprehensive Cancer Center, Keck School of Medicine, University of Southern California, Los Angeles, CA 90089, USA; ^4^Department of Biomedical Engineering, University of Southern California, Los Angeles, CA 90089, USA; ^5^Department of Pharmacology and Pharmaceutical Sciences, University of Southern California, Los Angeles, CA 90089, USA

## Abstract

Targeting nanoparticles by conjugating various specific ligands has shown potential therapeutic efficacy in nanomedicine. However, poor penetration of antitumor drugs into solid tumors remains a major obstacle. Here, we describe a targeting strategy for antitumor drug delivery by conjugating a crosslinked multilamellar liposomal vesicle (cMLV) formulation with a tumor-penetrating peptide, iRGD. The results showed that iRGD peptides could facilitate the binding and cellular uptake of drug-loaded cMLVs and consequently enhance the antitumor efficacy in breast tumor cells, including multidrug-resistant cells. Moreover, colocalization data revealed that iRGD-conjugated cMLVs (iRGD-cMLVs) entered cells via the clathrin-mediated pathway, followed by endosome-lysosome transport for efficient drug delivery. Finally, *in vivo* study indicated that iRGD-cMLVs could deliver anticancer drugs efficiently to mediate significant tumor suppression.

## 1. Introduction

For optimal anticancer treatment with cytotoxic drugs, it is necessary to sustain antitumor effects over a prolonged period at an efficacious drug concentration without inducing severe systemic toxicity. Therefore, as an alternative to conventional medicine for cancer therapeutics, nanoparticle-based drug delivery systems have been widely evaluated and utilized to modulate the toxicity profile of anticancer drugs and improve drug circulation time [1–3]. Long-circulating liposomes, such as polyethylene-glycol-(PEG-) coated liposomes, have become one of the most popular nanocarriers for delivering therapeutics and have shown the ability to passively accumulate in tumors as a result of enhanced permeability and retention (EPR) effect [4, 5]. Ultimately, however, active targeting to tumor cells via the inclusion of a tumor-targeting molecule on the nanocarriers is expected to provide more effective cancer therapy [1, 6, 7]. Once extravasated in the tumor environment, the targeting molecules will likely foster the active attachment of nanoparticles to tumor cells expressing the specific receptors for elevated antitumor activity.

Scientific investigations have identified diverse tumor-targeting molecules that can be exploited by nanoparticles to actively target cancer cell-specific markers with unique phenotypes in tumors. For example, it has been reported that drug carriers conjugated with targeting ligands, such as anti-Her2 antibody [8], folate [9], or transferrin (Tf) [10], have achieved therapeutic benefit by successfully targeting human epidermal receptors (HER), folate receptors, and transferrin receptor (TfR), respectively, all of which are overexpressed on tumor cells. The cell- or tissue-specific ligand-receptor interaction contributes to the increased efficacy as a result of enhanced uptake of the complex into tumor cells by receptor-mediated endocytosis. However, a major obstacle against the clinical application of this targeting strategy has been the poor penetration of the targeted payload through the vascular wall and into the tumor parenchyma, especially in solid tumors, which have a high interstitial pressure [11, 12]. Recently, a tumor-penetrating peptide, iRGD (CRGDKGPDC), was identified and reported to increase vascular and tissue penetration in a tumor-specific and neuropilin-1-dependent manner, as compared to conventional RGD peptides [13, 14]. Like conventional RGD peptides, iRGD homes to tumor sites by binding to *α*_*v*_ *β*_3_ and *α*_*v*_ *β*_5_ integrins, which are highly expressed in tumor endothelium [13, 15, 16], thus enhancing the therapeutic effect of antitumor drugs on suppressing tumor growth and/or metastasis. After binding, the iRGD peptide is thought to be proteolytically cleaved to produce CRGDK fragment, which favors binding to neuropilin-1 receptor, thus facilitating the penetration of drugs into the tumor [17]. 

Here, we explored whether the iRGD peptide could enhance cancer drug delivery and antitumor activity when conjugated to liposomal nanoparticles. Our previous studies evaluated nanoparticles based on a crosslinked multilamellar liposomal vesicle (cMLV), and we found that they exhibited remarkable stability, sustained release kinetics of encapsulated doxorubicin, and improved therapeutic efficiency *in vivo* [18]. Therefore, in this study, we tested the hypothesis that cMLV nanoparticles conjugated with iRGD peptides could enhance the delivery of the antitumor drug doxorubicin. We demonstrated that iRGD could increase both binding and uptake of Dox-loaded cMLV in 4T1 tumor cells. Moreover, the colocalization data showed that iRGD peptides could change the intracellular endocytic routes of cMLV particles, which was further confirmed by the drug-inhibition experiment. Data also showed that systemic injection of iRGD-conjugated nanoparticles could more efficiently suppress tumor growth in the breast tumor model. These results confirmed that the tumor-penetrating peptide iRGD could be a promising means of targeted drug delivery to tumor sites.

## 2. Materials and Methods

### 2.1. Materials

*Mice.* Female 6- to 10-week-old BALB/c mice were purchased from Charles River Breeding Laboratories (Wilmington, MA). All mice were held under specific pathogen-reduced conditions in the Animal Facility of the University of Southern California (USA). All experiments were performed in accordance with the guidelines set by the National Institutes of Health and the University of Southern California on the Care and Use of Animals.

*Cell Lines, Antibodies, and Reagents.* 4T1 tumor cells (ATCC number: CRL-2539) and JC cells (ATCC number: CRL-2116) were maintained in a 5% CO_2_ environment with Dulbecco's modified Eagle's medium (Mediatech, Inc., Manassas, VA) supplemented with 10% FBS (Sigma-Aldrich, St. Louis, MO) and 2 mM of L-glutamine (Hyclone Laboratories, Inc., Omaha, NE). The mouse monoclonal antibodies against clathrin, caveolin-1, and EEA1 were purchased from Santa Cruz Biotechnology, Inc. (Santa Cruz, CA). The mouse monoclonal antibody to Lamp-1 was purchased from Abcam (Cambridge, MA). Alexa488-TFP ester and Alexa488-goat anti-mouse immunoglobulin G (IgG) were obtained from Invitrogen (Carlsbad, CA). Chlorpromazine (CPZ) and Filipin were obtained from Sigma-Aldrich (St. Louis, MO) and used at appropriate concentrations according to the manufacturer's protocols.

### 2.2. Synthesis of iRGD-cMLVs

 Preparation of liposomes was based on the conventional dehydration-rehydration method. All lipids were obtained from NOF Corporation (Japan). 1.5 *μ*mol of lipids 1,2-dioleoyl-sn-glycero-3-phosphocholine (DOPC), 1,2-dioleoyl-sn-glycero-3-phospho-(1′-rac-glycerol) (DOPG), and maleimide-headgroup lipid 1,2-dioleoyl-sn-glycero-3-phosphoeth-anolamine-N-[4-(p-maleimidophenyl) butyramide (MPB-PE) were mixed in chloroform to form a lipid composition with a molar ratio of DOPC : DOPG : MPB = 4 : 1 : 5, and the organic solvent in the lipid mixture was evaporated under argon gas, followed by additional drying under vacuum overnight to form dried thin lipid films. The resultant dried film was hydrated in 10 mM Bis-Tris propane at pH 7.0 with doxorubicin at a molar ratio of 0.2 : 1 (drugs : lipids) with vigorous vortexing every 10 min for 1 h and then applied with 4 cycles of 15 s sonication (Misonix Microson XL2000, Farmingdale, NY) on ice at 1 min intervals for each cycle. To induce divalent-triggered vesicle fusion, MgCl_2 _was added to make a final concentration of 10 mM. The resulting multilamellar vesicles were further crosslinked by addition of dithiothreitol (DTT, Sigma-Aldrich) at a final concentration of 1.5 mM for 1 h at 37°C. The resulting vesicles were collected by centrifugation at 14,000 g for 4 min and then washed twice with PBS. For iRGD conjugation to cMLVs, the particles were incubated with 0.5 *μ*mol of iRGD peptides (GenScript, Piscataway, NJ) for 1 h at 37°C. For pegylation of cMLVs, both unconjugated and iRGD-conjugated particles were further incubated with 0.5 *μ*mol of 2 kDa PEG-SH (Laysan Bio Inc., Arab, AL) for 1 h at 37°C. The particles were then centrifuged and washed twice with PBS. The final products were stored in PBS at 4°C.

### 2.3. Characterization of Physical Properties

 The hydrodynamic size and size distribution of iRGD-cMLVs were measured by dynamic light scattering (Wyatt Technology, Santa Barbara, CA).

### 2.4. **In Vitro** Drug Encapsulation and Release

 To study the loading capacity of Dox, iRGD-cMLV(Dox) nanoparticles were collected and then washed twice with PBS, followed by lipid extraction of vesicles with 1% Triton X-100 treatment. Dox fluorescence (excitation 480 nm, emission 590 nm) was then measured by a Shimadzu RF-5301PC spectrofluorometer (Japan). To obtain the release kinetics of Dox from liposomes, Dox-loaded iRGD-cMLVs were incubated at 37°C in 10% fetal-bovine-serum-(FBS-) containing media, the releasing media were removed from iRGD-cMLVs incubated at 37°C for quantification of Dox fluorescence every day, and fresh media were replaced for continuous monitoring of drug release.

### 2.5. **In Vitro** Cytotoxicity

4T1 and JC cells were plated at a density of 5 × 10^3^ cells per well in D10 media in 96-well plates and grown for 6 h. The cells were then exposed to a series of concentrations of cMLV(Dox) or iRGD-cMLV(Dox) for 48 h, and the cell viability was assessed using the Cell Proliferation Kit II (XTT assay) from Roche Applied Science (Indianapolis, IN) according to the manufacturer's instructions. Cell viability percentage was determined by subtracting absorbance values obtained from media-only wells from drug-treated wells and then normalizing to the control cells without drugs. The data were analyzed by nonlinear regression to get the IC_50_ value.

### 2.6. **In Vitro** Binding and Internalization Study

4T1 cells were plated at a density of 2 × 10^5^ cells per well in D10 media in 24-well plates and grown overnight. The cells were incubated with two concentrations (0.2 *μ*g/mL and 0.04 *μ*g/mL) of iRGD-cMLV(Dox) or cMLV(Dox) for 30 min at 4°C (for binding assay) or 2 h at 37°C (for internalization assay). After incubation, the cells were washed twice with PBS to remove the unbound nanoparticles. Binding and cellular uptake of particles were determined by measuring doxorubicin fluorescence using flow cytometry.

### 2.7. Confocal Imaging

Fluorescence images were acquired on a Yokogawa spinning-disk confocal scanner system (Solamere Technology Group, Salt Lake City, UT) using a Nikon eclipse Ti-E microscope equipped with a 60 × /1.49 Apo TIRF oil objective and a Cascade II: 512 EMCCD camera (Photometrics, Tucson, AZ, USA). An AOTF (acousto-optical tunable filter) controlled laser-merge system (Solamere Technology Group Inc.) was used to provide illumination power at each of the following laser lines: 491 nm, 561 nm, and 640 nm solid state lasers (50 mW for each laser).

To label liposomal particles, DiD lipophilic dyes were added to the lipid mixture in chloroform at a ratio of 0.01 : 1 (DiD : lipids), and the organic solvent in the lipid mixture was evaporated under argon gas to incorporate DiD dyes into a lipid bilayer of vesicles. To detect iRGD peptides, both iRGD-cMLV and unconjugated cMLV particles were incubated with 50 nmol of Alexa488-TFP ester (Invitrogen) for 2 h in 0.1 M sodium bicarbonate buffer (pH = 9.3). After 2 h incubation, the reaction was stopped, and unbound dye molecules were removed via buffer exchange into PBS (pH = 7.4) using a Zeba desalting spin column (Fisher Scientific). For the detection of intracellular nanoparticles, DiD-labeled iRGD-cMLV or DiD-labeled unconjugated cMLV were incubated for 30 min at 4°C with HeLa cells that were seeded overnight on polylysine-coated glass bottom dishes (MatTek Corporation, Ashland, MA). Then the samples were incubated at 37°C to initiate particle internalization at the indicated time points. The culture dish was then rinsed, fixed with 4% formaldehyde, permeabilized with 0.1% Triton X-100, and then immunostained with the corresponding antibodies specific to clathrin, caveolin-1, EEA1, or Lamp-1 and counterstained with DAPI (Invitrogen, Carlsbad, CA).

### 2.8. Uptake Inhibition Assay

HeLa cells (1 × 10^5^ cells) were preincubated with Chlorpromazine (CPZ, 25 *μ*g/mL) or Filipin (10 *μ*g/mL) for 30 min to disrupt the clathrin- or caveolin-mediated pathway. The cells were then incubated with DiD-labeled iRGD-cMLV or unconjugated cMLV for 1 h at 37°C in the presence of CPZ and filipin. The cells were then washed twice with PBS. The cellular uptake of particles was determined by measuring DiD fluorescence using flow cytometry and normalized on the basis of fluorescent intensity acquired from the untreated cells.

### 2.9. **In Vivo** Antitumor Activity Study

BALB/c female mice (6–10 weeks old) were inoculated subcutaneously with 0.2 × 10^6^ 4T1 breast tumor cells. The tumors were allowed to grow to a volume of ~50 mm^3^ before treatment. On day 10, the mice were injected intravenously through tail vein with PBS (control group), cMLV (2 mg/kg Dox), and iRGD-cMLV (2 mg/kg Dox) every three days (five mice per group). Tumor growth and body weight were then monitored until the end of the experiment. The length and width of the tumor masses were measured with a fine caliper every three days after injection. Tumor volume was expressed as 1/2 × (length × width^2^).

## 3. Results

### 3.1. Preparation of iRGD-cMLV Nanoparticles

**Figure 1:**
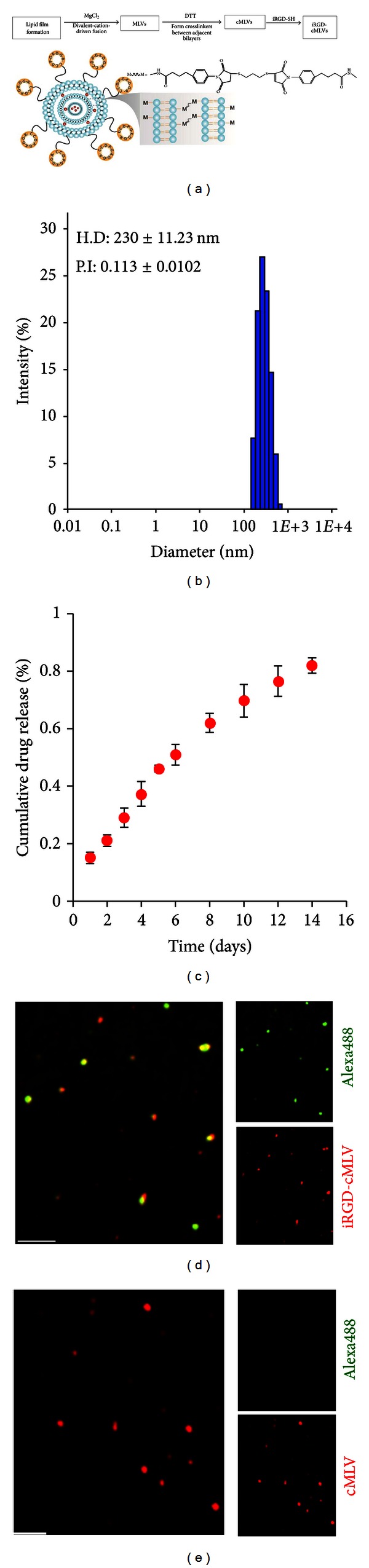
Characterization, release profile, and conjugation of iRGD-cMLVs. (a) Schematic illustration of the synthesis of iRGD-conjugated crosslinked multilamellar vesicle (iRGD-cMLV). (b) The hydrodynamic size distribution of iRGD-cMLVs measured by dynamic light scattering (DLS). Data represented the mean ± SD of at least three experiments with *n* = 3. (c) *In vitro* release kinetics of doxorubicin (Dox) from iRGD-cMLVs. Error bars represent standard error of the mean; *n* = 3 for each formulation. ((d), (e)) Confirmation of the conjugation of iRGD peptides onto the cMLV nanoparticles by confocal imaging. DiD-labeled iRGD-cMLVs (d) and DiD-labeled cMLVs (e) were reacted with Alexa488 dye for 1 h at room temperature followed by confocal imaging. Scale bar represents 5 *μ*m.

The procedure for the preparation of crosslinked multilayer liposomal vesicles (cMLV) was adapted from a recently reported multistep procedure based on the conventional dehydration-rehydration method to form covalent crosslinkers between adjacent lipid bilayers [19], as illustrated in Figure 1(a). This method employed a divalent cation-triggered vesicle fusion to yield a multilamellar structure, from which interbilayer crosslinkers were formed across the opposing sides of lipid bilayers through the reactive headgroups with dithiothreitol (DTT). The iRGD peptides (CRGDKGPDC) were conjugated to the surface of cMLVs through the functional thiol-reactive maleimide headgroups of maleimide-headgroup lipid, 1,2-dioleoyl-sn-glycero-3-phosphoeth-anolamine-N- [4-(p-maleimidophenyl) butyramide](MPB-PE). As a final step, the surface of the iRGD-conjugated cMLV (iRGD-cMLV) was pegylated with thiol-terminated PEG to further improve the blood circulation time of vesicles [5, 20].

The physical properties of synthesized iRGD-cMLV were characterized. The hydrodynamic size of these targeted nanoparticles was measured by dynamic light scattering (DLS), and the result showed the mean diameter of iRGD-cMLV to be ~230 ± 11.23 nm (Figure 1(b)), which was similar to that of unconjugated cMLV (~220 ± 6.98 nm). Moreover, it has been confirmed that doxorubicin-(Dox-) encapsulation efficiency of ~85% can be achieved via this preparation procedure. An *in vitro* drug release assay also showed that iRGD-cMLV exhibited slow and sustained release kinetics (up to 2 weeks) in a serum environment (Figure 1(c)).

Next, we examined whether iRGD peptides were conjugated to the surface of cMLV via the maleimide headgroups. To this end, fluorescent 1,1-dioctadecyl-3,3,3,3-tetramethylindodicarbocyanine-(DiD-) labeled cMLV particles were used to visualize both unconjugated and conjugated particles. In addition, Alexa488 dye was utilized to label iRGD peptides through the amine group of lysine residues on iRGD peptides (CRGD**K**GPDC). The results showed that a significant colocalization of DiD-labeled iRGD-cMLV particles with Alexa488-labeled iRGD peptides was observed (Figure 1(d)), while no Alexa488 signals were detected on unconjugated cMLV particles (Figure 1(e)), suggesting that iRGD peptides were successfully conjugated to cMLV particles.

### 3.2. Cytotoxicity and Cell Uptake of iRGD-cMLV(Dox)

**Figure 2:**
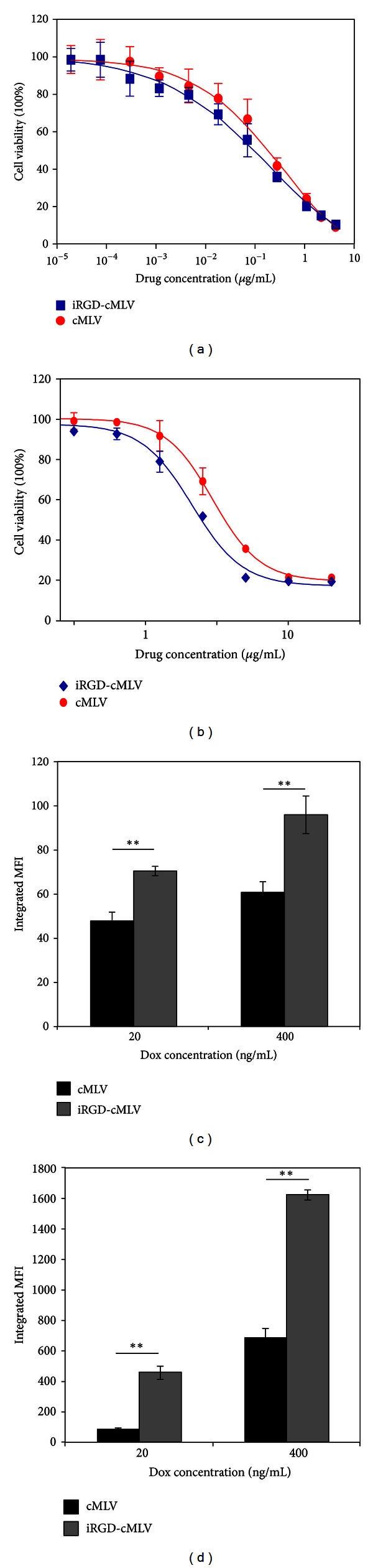
*In vitro* cytotoxicity, binding, and internalization of iRGD-cMLVs and cMLVs in tumor cells. ((a), (b)) *In vitro* cytotoxicity of cMLV(Dox) and iRGD-cMLV(Dox) in 4T1 tumor (a) and multidrug-resistant JC cells (b). The cytotoxicity was measured by a standard XTT assay. Error bars represent the standard deviation of the mean from triplicate experiments. ((c), (d)) Binding and internalization of cMLV(Dox) and iRGD-cMLV(Dox) to 4T1 cells. 4T1 cells were incubated with cMLV(Dox) and iRGD-cMLV(Dox) for 30 min at 4°C (c) or 2 h at 37°C (d). Both binding and cellular uptake of nanoparticles were determined by measuring doxorubicin fluorescence using flow cytometry. Statistical analysis was performed with Student's *t*-test. Error bars represent the standard deviation of the mean from triplicate experiments.

We next determined the effect of iRGD-conjugated cMLV nanoparticles on cytotoxicity levels in cells as compared to unconjugated cMLV nanoparticles. Dox-loaded cMLV (cMLV(Dox)) and Dox-loaded iRGD-cMLV (iRGD-cMLV(Dox)) were incubated with 4T1 or JC cells. JC cells represent a model drug-resistant tumor cell line overexpressing P-glycoprotein and exhibiting drug-resistant phenotype both *in vitro* and *in vivo *[21]. After 48 h incubation, the cytotoxicity of Dox liposomes was measured by a standard XTT assay. *In vitro* cytotoxicity data revealed that iRGD-cMLV showed slightly smaller IC_50_ (0.011 ± 0.0037 *μ*g/mL) in 4T1 cells as compared to cMLV (0.018 ± 0.0025 *μ*g/mL) (Figure 2(a)). A significant difference of cytotoxicity between iRGD-cMLV(Dox) and cMLV(Dox) was observed in JC cells, in which iRGD-cMLV(Dox) showed a lower IC_50_ (2.01 ± 0.22 *μ*g/mL) value than that of cMLV(Dox) (3.19 ± 0.32 *μ*g/mL, *P* < 0.05, Figure 2(b)). The XTT results indicated that delivery of Dox with iRGD-conjugated cMLV was more potent in inhibiting tumor cell proliferation.

To investigate whether the enhanced cell cytotoxicity of iRGD-cMLV resulted from an increased cellular uptake of nanoparticles, the cellular binding and uptake of iRGD-cMLV and cMLV were examined. For the binding assay, cMLV(Dox) or iRGD-cMLV(Dox) was incubated with 4T1 tumor cells at 4°C for 30 min. Then the bound nanoparticles on the cell surface were determined by detecting doxorubicin signals via flow cytometry after removing the unbound nanoparticles. As shown in Figure 2(c), at both concentrations, a significantly higher integrated mean fluorescence intensity (MFI) was observed when the cells were incubated with iRGD-cMLV(Dox), indicating that iRGD-cMLVs can facilitate the attachment of nanoparticles to the cells via the integrin receptor expressed on the surface of tumor cells (*P* < 0.01). Additionally, the cellular accumulation of doxorubicin in 4T1 cells was determined by integrated MFI after the cells were incubated with cMLV(Dox) or iRGD-cMLV(Dox) at 37°C for 2 h. The results showed that a remarkably enhanced cell uptake of doxorubicin was observed when the cells were incubated with iRGD-cMLV(Dox) (*P* < 0.01, Figure 2(d)), suggesting that the increased cellular accumulation of doxorubicin was facilitated by iRGD peptides. Taken together, the iRGD peptides promoted both binding and uptake of drug-loaded nanoparticles in tumor cells, thereby enhancing the drug concentration in cells and improving the cytotoxicity of drugs.

### 3.3. Internalization and Intracellular Pathways of iRGD-cMLVs

**Figure 3:**
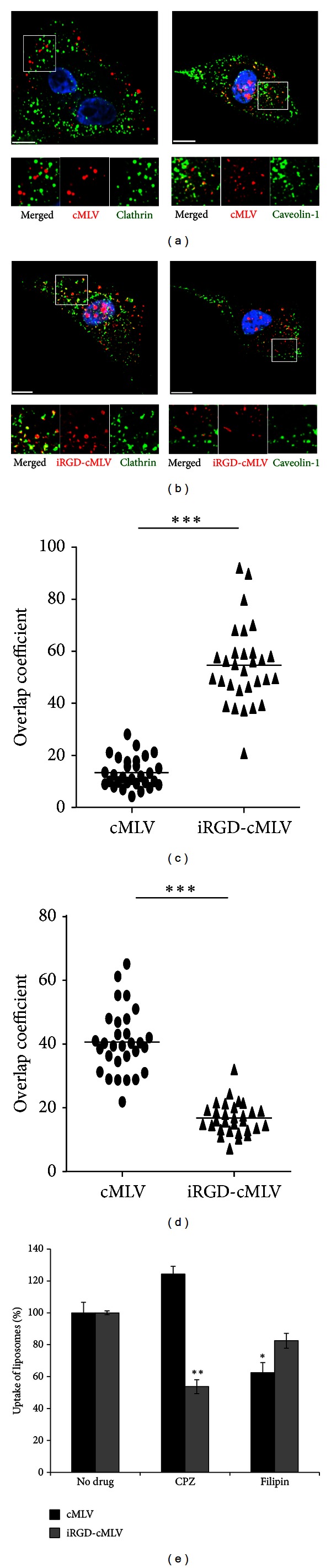
Clathrin-mediated internalization of iRGD-cMLVs and caveolin-dependent endocytosis of cMLVs. ((a), (b)) HeLa cells were incubated with DiD-labeled cMLV nanoparticles (red, (a)) or DiD-labeled iRGD-cMLVs particles (red, (b)) for 30 min at 4°C to synchronize internalization. The cells were then incubated at 37°C for 15 min, fixed, permeabilized, and immunostained with anti-clathrin (green) or anti-caveolin-1 antibody (green). The nucleus of cells was counterstained with DAPI. Scale bar represents 10 *μ*m. ((c), (d)) Quantification of cMLV and iRGD-cMLV particles colocalized with clathrin (c) or caveolin-1 signals (d) after 15 min of incubation. Overlap coefficients were calculated using Manders' overlap coefficients by viewing more than 30 cells of each sample using the Nikon NIS-Elements software. Error bars represent the standard deviation of the mean from analysis of multiple images (***P < 0.005). (e) Inhibition of clathrin-dependent endocytosis by chlorpromazine (CPZ, 25 *μ*g/mL) and caveolin-dependent internalization by Filipin (10 *μ*g/mL). The uptake of DiD-labeled cMLV and DiD-labeled iRGD-cMLV nanoparticles was determined by measuring DiD fluorescence via flow cytometry. Error bars represent the standard deviation of the mean from triplicate experiments (*P < 0.05, **P < 0.01).

We next investigated the entry mechanism and intracellular process of iRGD-cMLV into tumor cells to determine whether iRGD peptides could change the pathway by which nanoparticles are endocytosed. Endocytosis is known as one of the main entry mechanisms for various nanoscale drug carriers [22, 23]. Several studies have reported the involvement of clathrin- and caveolin-dependent pathways in nanoparticle-mediated endocytosis [24–26]. Therefore, to investigate the role of clathrin- or caveolin-dependent endocytosis of iRGD-cMLVs, we visualized the individual fluorescent DiD-labeled cMLVs or iRGD-cMLVs with endocytic structures (clathrin or caveolin) after 15 min incubation at 37°C. As shown in Figure 3(a), a significant colocalization of unconjugated cMLV particles with caveolin-1 signals was observed, while no colocalization between unconjugated cMLV particles and clathrin structures was detected, indicating that the caveolin pathway may be involved in the endocytosis of cMLVs. However, after 15 min incubation, iRGD-cMLV particles were colocalized with clathrin structures, whereas, no significant colocalization between iRGD-cMLV particles and caveolin-1 signals was observed (Figure 3(b)), suggesting that the endocytosis of iRGD-cMLVs could be clathrin dependent. The quantification of iRGD-cMLVs and cMLVs colocalized with caveolin-1 or clathrin structures by analyzing more than 30 cells confirmed that the clathrin-mediated pathway could be involved in the entry of iRGD-cMLVs, while the endocytosis of cMLVs could be caveolin-1 dependent (Figures 3(c) and 3(d)). The role of clathrin-dependent endocytosis of iRGD-cMLV was further examined by drug-inhibition assays shown in Figure 3(e). Chlorpromazine (CPZ) is known to block clathrin-mediated internalization by inhibiting clathrin polymerization [27], while filipin is a cholesterol-binding reagent that can disrupt caveolin-dependent internalization [28, 29]. As shown in Figure 3(e), CPZ (10 *μ*g/mL) significantly decreased the uptake of iRGD-cMLV particles in HeLa cells, while no significant inhibitory effect on their uptake was observed when cells were pretreated with Filipin (10 *μ*g/mL). However, pretreatment of cells with Filipin remarkably decreased the uptake of unconjugated cMLV particles (*P* < 0.01), whereas no inhibitory effect on their uptake was observed in CPZ-pretreated cells. Results from the inhibition assay further confirmed that iRGD-cMLV endocytosis is mediated by the clathrin-dependent pathway, while unconjugated cMLV particles enter cells via caveolin-dependent endocytosis.

**Figure 4:**
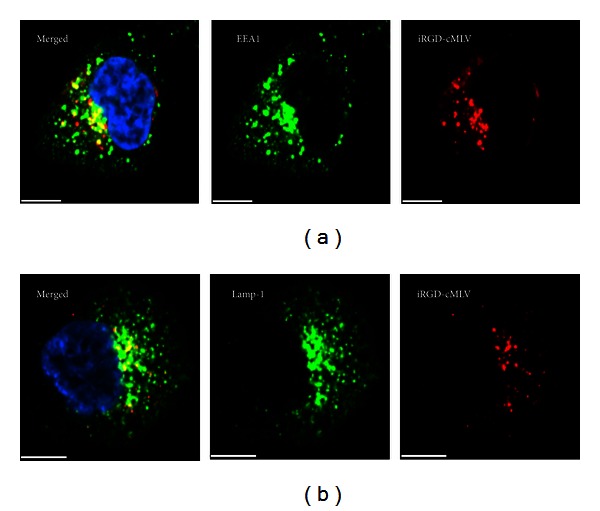
Involvement of early endosomes and lysosomes in the intracellular trafficking of iRGD-cMLVs. HeLa cells were incubated with DiD-labeled iRGD-cMLV nanoparticles (red) for 30 min at 4°C to synchronize internalization. The cells were then incubated at 37°C for 45 min and immunostained with anti-EEA1 antibody (green, (a)) or for 2 h and immunostained with anti-Lamp1 antibody (green, (b)). The nucleus of cells was counterstained with DAPI. Scale bar represents 10 *μ*m.

Once inside the cells, the intracellular fate of the endosomal contents has been considered as an important determinant of successful drug delivery [30]. It was also proposed that nanoparticles might transport to the early endosomes in a GTPase Rb5-dependent manner and also proceed through the conventional endocytic pathway (endosomes/lysosomes) [31–33], probably resulting in enzymatic destruction of lipid membrane for drug release in lysosomes [30]. To further investigate the subsequent intracellular fate of iRGD-cMLV nanoparticles, DiD-labeled iRGD-cMLV particles were evaluated for their colocalization with the early endosome (EEA-1) [34] and lysosome (Lamp-1) [31] markers at different incubation times at 37°C. As shown in Figure 4(a), most iRGD-cMLV particles were found in the EEA1^+^ early endosomes after incubation of 30 min, validating the involvement of early endosomes in the intracellular fate of targeted cMLV particles. In addition, after 2 h incubation, a significant colocalization of iRGD-cMLVs with lysosomes was observed, suggesting that iRGD-cMLVs may transport to early endosomes and further travel to lysosomes for possible release of drug from liposomes and endocytic compartments to cytosol. When taken together, the results showed that iRGD-cMLVs enter tumor cells via clathrin-dependent and receptor-mediated endocytosis, followed by transport through early endosomes and lysosomes.

### 3.4. Therapeutic Effect of iRGD-cMLV(Dox) in Breast Tumor Animal Model

**Figure 5:**
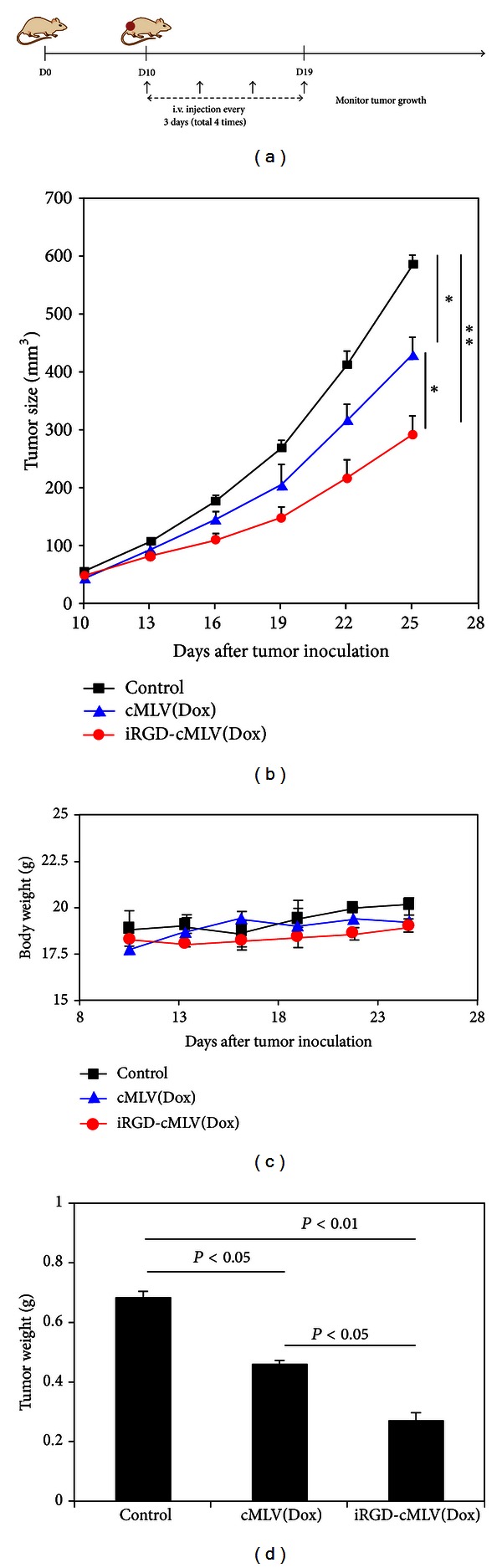
Antitumor effect of iRGD-cMLVs and cMLVs in the 4T1 breast tumor model. (a) Schematic diagram of the experimental protocol for the *in vivo* tumor study. (b) Tumor growth was measured after treatment without injection (control), cMLV(Dox), and iRGD-cMLV(Dox) (2 mg/kg Dox equivalents). Error bars represent standard error of the mean; *n* = 5 for each treatment group (*P < 0.05). (c) Average mouse weight loss over the duration of the experiment. (d) Tumor weight of excised tumors from each treatment group at 25 days after tumor inoculation. Error bars represent standard error of the mean; *n* = 5 for each treatment group.

We have demonstrated that iRGD-conjugated cMLVs can enhance uptake of nanoparticles into cells, resulting in an increased concentration of doxorubicin and *in vitro* cytotoxicity. Here, a breast tumor animal model was used to evaluate the *in vivo* therapeutic efficacy of iRGD-cMLV(Dox), compared with that of cMLV(Dox). At day 0, BALB/c mice were inoculated subcutaneously with 4T1 breast tumor cells. At day 10, mice were injected intravenously with iRGD-cMLV(Dox) or cMLV(Dox) at doses of 2 mg/kg Dox equivalents every three days. Tumor growth and body weight were then monitored until the end of the experiment (Figure 5(a)). As shown in Figure 5(b), mice in the group receiving 2 mg/kg cMLV(Dox) showed a significant tumor inhibition as compared to mice in the untreated group (*P* < 0.01). In addition, a marked suppression of tumor growth was observed in the group treated by iRGD-cMLV(Dox), suggesting that iRGD peptides could further enhance the therapeutic effect of drug-loaded nanoparticles *in vivo*. During the whole experiment, no weight loss was seen in any of the mice (Figure 5(c)), indicating the absence of systemic toxicity from cMLV and iRGD-cMLV formulations. The enhanced antitumor activity of iRGD-cMLV (Dox) was further confirmed by a significant reduction on tumor weight of mice treated with iRGD-cMLV(Dox), as compared to that treated with cMLV(Dox) (Figure 5(d)).

## 4. Discussion

Nontargeted, long-circulating liposomes, such as Doxil/Caelyx, have been extensively evaluated to deliver chemotherapeutic drugs to treat cancers via the enhanced permeability and retention mechanism [35–37]. Although significant efforts have been made to enhance their therapeutic activity, the relatively inherent instability of conventional liposomes in the presence of serum component, resulting in rapid drug release profile, has been considered as an obstacle in their development for cancer treatment [38]. In order to develop a liposomal formulation with sustainable release kinetics and improved stability, a cMLV formulation of Dox has been explored as a new nanocarrier platform with promising features of enhanced vesicle stability and reduced systemic toxicity, resulting in improved *in vivo* therapeutic efficiency [18]. Although cMLVs have shown improved antitumor activity, direct delivery of these particles with targeting ligands could potentially further enhance efficacy and minimize toxicity.

Most currently investigated targeting strategies concentrate on directing nanoparticles to tumor cells by utilizing the specific receptor/ligand overexpressed on tumor cells [6, 39, 40]. For instance, RGD (arginine-glycine-aspartate) peptides have been conjugated to drug-loaded nanoparticles to target integrin receptors, which are overexpressed on neovascular endothelial cells [13, 15, 16]. Although the development of targeted payload for anticancer drug delivery has shown potential enhanced therapeutic effect, poor penetration of nanoparticles to tumor cells still thwarts clinical treatment of solid tumor [11, 12]. Therefore, a novel iRGD peptide has been recently identified and reported to increase vascular and tissue penetration in a tumor-specific and neuropilin-1- (NRP-1-) dependent manner [13, 15, 16]. The C-terminal motif CendR of iRGD peptide has been identified as a mediator of cell and tissue penetration through the interaction with neuropilin-1 receptor, a cell-surface receptor that is involved in the regulation of vascular permeability [41, 42]. For example, it has been reported that the successful infection of many viruses required proteolytic cleavage of capsid proteins to expose the CendR motifs to neuropilin-1 receptor, which could trigger the endocytosis of viral particles into cells [43]. Moreover, several studies have reported that peptides containing CendR motifs could bind to NRP-1 receptor and cause cellular internalization and vascular leakage [44], suggesting that iRGD peptides could have similar effects when covalently coupled to a drug delivery nanocarrier. Previously, we demonstrated the enhanced therapeutic ability of cMLV formulations with reduced systemic toxicity, as compared to that of unilamellar liposome or Doxil-like liposomes [18]. Therefore, in this study, we conjugated iRGD peptides to this relatively stable cMLV particles and evaluated, both *in vitro* and *in vivo*, the effect of these targeted nanoparticles. A similar accumulative drug release profile was observed in iRGD-cMLV formulation as compared to cMLV formulations, due to a similar size distribution and lipid composition of these two formulations. The results showed that iRGD-cMLVs presented superior cytotoxicity resulting from the enhanced binding and uptake of targeted nanoparticles in cells. Moreover, enhanced uptake and penetration of Dox via iRGD-cMLV vesicles enabled the improved *in vivo* therapeutic activity in tumors. iRGD-cMLVs treatment of 4T1 tumors exhibited significant inhibition of tumor growth compared to that treated with cMLVs, further suggesting the potential application of iRGD to drug delivery via nanoparticles.

Furthermore, our imaging study on the entry mechanism of iRGD-cMLVs provided some edifying details about the intracellular fate of these particles. Specifically, the results showed that iRGD-cMLV particles enter cells via clathrin-dependent endocytosis, while the internalization of unconjugated cMLV particles is caveolin-mediated. The different endocytic pathways utilized by iRGD-cMLVs might result from the interaction of nanoparticles with cells via iRGD-integrin binding. The results also suggested that the receptor mediated internalization possibly promoted cell attachment, resulting in an enhanced cellular uptake. Although it has been hypothesized that multiple pathways were involved in endosomal transport [24, 25, 45], our data showed that both iRGD-cMLVs and cMLVs home to early endosomes and further traffic to lysosomes [18]. The involvement of lysosome in the intracellular trafficking routes of both iRGD-cMLVs and cMLVs might facilitate drug release kinetics because enzymes, such as phospholipases, in the endolysosomal compartments can promote disruption of liposomal bilayers [46, 47].

## 5. Conclusions

This study has evaluated the potential therapeutic effects of a tumor-penetrating peptide, iRGD, by conjugating it with Dox-loaded cMLVs in tumor treatment. We have demonstrated that iRGD-cMLVs can serve as a new targeting strategy to facilitate the penetration of antitumor drugs into tumor cells and further enhance the therapeutic efficacy of drugs both *in vitro* and *in vivo*. In addition, the endocytic pathways involved in the entry of iRGD-cMLVs have been investigated to shed some light on the possible mechanism of enhanced cellular uptake of targeted nanoparticles.
